# Microcalcifications in breast cancer: an active phenomenon mediated by epithelial cells with mesenchymal characteristics

**DOI:** 10.1186/1471-2407-14-286

**Published:** 2014-04-23

**Authors:** Manuel Scimeca, Elena Giannini, Chiara Antonacci, Chiara Adriana Pistolese, Luigi Giusto Spagnoli, Elena Bonanno

**Affiliations:** 1 Anatomic Pathology Section, Department of Biomedicine and Prevention, University of Rome “Tor Vergata”, Via Montpellier 1, Rome 00133, Italy; 2 Diagnostic Imaging Section, Department of Biomedicine and Prevention, University of Rome “Tor Vergata”, Via Montpellier 1, Rome 00133, Italy

## Abstract

**Background:**

Mammary microcalcifications have a crucial role in breast cancer detection, but the processes that induce their formation are unknown. Moreover, recent studies have described the occurrence of the epithelial–mesenchymal transition (EMT) in breast cancer, but its role is not defined. In this study, we hypothesized that epithelial cells acquire mesenchymal characteristics and become capable of producing breast microcalcifications.

**Methods:**

Breast sample biopsies with microcalcifications underwent energy dispersive X-ray microanalysis to better define the elemental composition of the microcalcifications. Breast sample biopsies without microcalcifications were used as controls. The ultrastructural phenotype of breast cells near to calcium deposits was also investigated to verify EMT in relation to breast microcalcifications. The mesenchymal phenotype and tissue mineralization were studied by immunostaining for vimentin, BMP-2, β2-microglobulin, β-catenin and osteopontin (OPN).

**Results:**

The complex formation of calcium hydroxyapatite was strictly associated with malignant lesions whereas calcium-oxalate is mainly reported in benign lesions. Notably, for the first time, we observed the presence of magnesium-substituted hydroxyapatite, which was frequently noted in breast cancer but never found in benign lesions. Morphological studies demonstrated that epithelial cells with mesenchymal characteristics were significantly increased in infiltrating carcinomas with microcalcifications and in cells with ultrastructural features typical of osteoblasts close to microcalcifications. These data were strengthened by the rate of cells expressing molecules typically involved during physiological mineralization (i.e. BMP-2, OPN) that discriminated infiltrating carcinomas with microcalcifications from those without microcalcifications.

**Conclusions:**

We found significant differences in the elemental composition of calcifications between benign and malignant lesions. Observations of cell phenotype led us to hypothesize that under specific stimuli, mammary cells, which despite retaining a minimal epithelial phenotype (confirmed by cytokeratin expression), may acquire some mesenchymal characteristics transforming themselves into cells with an osteoblast-like phenotype, and are able to contribute to the production of breast microcalcifications.

## Background

Microcalcifications play a crucial role in early breast cancer diagnosis, the second leading cause of cancer death among women [1]. Approximately 50% of non-palpable breast cancers are detected by mammography exclusively through microcalcification patterns [2], revealing up to 90% of ductal carcinoma *in situ*[3]. Mammary microcalcifications are classified according to their mammographic morphology, i.e. density and distribution [4], and by their physical and chemical properties [5]. Type I calcifications are composed of calcium oxalate (CO), and are amber-colored, partially transparent, and form pyramidal structures with relatively planar surfaces. Type II calcifications are composed of calcium phosphate, mainly hydroxyapatite (HA); they are grey-white, opaque with ovoid or fusiform shapes and have irregular surfaces [5-7]. The mechanisms that induce the formation of microcalcifications in breast cancer are still unknown, and for a long period of time they have been considered a passive phenomenon [8]. Recently, it has been suggested that ectopic mineralization in pathological conditions might be regulated by mechanisms similar to those occurring in physiological conditions [9,10]. Calcification of bone during skeletal growth [11,12] is sustained by mineralization-competent cells that are mesenchymal in origin, for example osteoblasts and hypertrophic chondrocytes [13], by three different processes: matrix vesicle-mediated mineral initiation [14,15], nucleation of mineral crystal [16,17] and ectopic mineralization [18].

Epithelial–mesenchymal transition (EMT), a complex phenomenon in which epithelial cells lose their characteristic traits and gain several properties of mesenchymal cells, is believed to play a role in breast cancer [19-21] and presents different changes at both the genetic and molecular level. EMT starts with the loss of cell polarity and the dissolution of tight junctions, allowing the intermingling of apical and basolateral membrane components [22]. Phenotypically, EMT involves the loss of epithelial cell markers such as E-cadherin and cytokeratin, and the acquisition of mesenchymal markers such as vimentin and nuclear β-catenin [23].

The first issue that we addressed in this study concerned the relationship between the elemental composition of calcification and the breast lesion type. The second approach was oriented to investigate if microcalcifications are related to an active process mediated by epithelial cells that enables acquisition of mesenchymal characteristics mimicking physiological mineralization.

To better define the phenomenon of microcalcifications, we took advantage of morphological characterization and microanalytical techniques correlating breast lesion types with the fine elemental composition of minerals. Furthermore, to assess a possible role of epithelial cells in tissue mineralization, we explored the cellular phenotype by correlating morphological data with molecular markers revealed by immunohistochemistry.

## Methods

### Breast sample collection

In this retrospective study, we collected 86 breast diagnostic biopsies in total: 60 vacuum-assisted needle biopsies, six surgical biopsies performed on radiologically suspicious breast microcalcifications and 20 samples of breast diagnostic biopsies without microcalcifications. Our study protocol was approved by the “Policlinico Tor Vergata” independent ethical committee (reference number # 94.13).

### Histology

After fixation in 10% buffered formalin for 24 h, breast tissues were embedded in paraffin. Three-micrometer-thick sections were stained with hematoxylin and eosin (H & E) and the diagnostic classification was blindly performed by two pathologists [24].

### Tissue microarray (TMA)

For TMA construction, we utilized fragments of tissues left over the sampling procedures for diagnostic purpose. Areas of interest from 20 infiltrating carcinomas without microcalcifications (ICwm) were identified in corresponding H & E-stained sections and marked on the donor paraffin block. A 3-mm-thick core of the donor block was placed in the recipient master block of the Galileo TMA CK2500 (Brugherio, Milan, Italy). Three cores from different areas of the same tissue block were arrayed for each case (total amount of neoplastic cells not less than 1.500) [25].

### Immunohistochemistry

Paraffin sections of 4-μm-thick were cut both from diagnostic blocks and TMA, and were processed by the Bench Mark automatized system (Ventana, Tucson, AZ, USA). After pretreatment, sections were incubated with rabbit monoclonal anti-vimentin (clone V9; Ventana, Tucson, AZ, USA; pre-diluted) [26], rabbit monoclonal anti-bone morphogenic protein-2 (clone N/A; Novus Biologicals, Littleton, CO, USA; 1:500 diluted) [27], rabbit monoclonal anti-β2 microglobulin (clone N/A; Dako Denmark A/S, Glostrup Denmark; 1:100 diluted) [28], rabbit monoclonal anti-β-catenin (clone 14; Ventana, Tucson, AZ, USA; pre-diluted) [29] and rabbit monoclonal anti-osteopontin (clone N/A; Novus Biologicals, Littleton, CO, USA; 1:100 diluted) [30] antibodies. Reactions were revealed with an ultraView Universal DAB Detection Kit (Ventana, Tucson, AZ, USA). For dual color immunohistochemistry, sections were stained using the same automatized system. Briefly, 4-μm-thick sections were pre-treated with CC1 reagent (Ventana, Tucson, AZ, USA) for 30 min at 95°C and then incubated with primary rabbit monoclonal anti-pan cytokeratin antibody for 20 min (clone AE1/AE3/PCK26; Ventana, Tucson, AZ, USA; pre-diluted). Reactions were revealed using an ultraView Universal DAB Detection Kit (Ventana, Tucson, AZ, USA). Sections were newly pre-treated with CC1 Ventana reagent for 8 min at 95°C and incubated with primary rabbit monoclonal anti-vimentin for 30 min (clone V9; Ventana, Tucson, AZ, USA). Vimentin reactions were revealed with an ultraView Universal Alkaline Phosphatase Red Detection Kit (Ventana, Tucson, AZ, USA).

### Transmission electron microscopy (TEM)

Small pieces of breast tissue from surgical specimens were fixed in 4% paraformaldehyde, post-fixed in 2% osmium tetroxide [31] and embedded both in EPON resin and in London ResinWhite (LR-White) resin for morphological and immunoultrastructural studies. After washing with 0.1 M phosphate buffer, the sample was dehydrated by a series of incubations in 30%, 50% and 70%, ethanol. For EPON resin, dehydration was continued by incubation steps in 95% ethanol, absolute ethanol and propylene oxide, then samples were embedded in Epon (Agar Scientific, Stansted Essex CM24 8GF United Kingdom) [32].

For LR-White embedding (Agar Scientific, Stansted Essex CM24 8GF United Kingdom), dehydration was completed with incubations in 70% ethanol–LR-White mixture (1:1) and LR-White absolute, then samples were embedded in LR-White resin [33]. After both types of incubation, tissues were cut [34,35] and stained with heavy metals solutions as described by Reynolds [36].

### Energy dispersive x-ray (EDX) microanalysis

All breast samples underwent ultrastructural microanalysis. Six-micrometer-thick paraffin sections were embedded in Epon resin following identification of microcalcifications. Briefly, sections were deparaffinized, hydrated, osmium tetroxide-fixed, dehydrated in ethanol and propylene oxide and infiltrated in Epon. The embedding capsules were positioned over areas containing previously-identified microcalcifications. Unstained ultra-thin sections of approximately 100-nm-thick were mounted on copper grids for microanalysis. EDX spectra of microcalcifications were acquired with a Hitachi 7100FA transmission electron microscope (Hitachi, Schaumburg, IL, USA) and an EDX detector (Thermo Scientific, Waltham, MA, USA) at an acceleration voltage of 75 KeV and magnification of 12.000. Spectra were semi-quantitatively analyzed by the Noram System Six software (Thermo Scientific, Waltham, MA, USA) using the standardless Cliff-Lorimer k-factor method [37]. EDX microanalysis apparatus was calibrated using an x-ray microanalysis standard (Micro-Analysis Consultants Ltd., Cambridgeshire, UK).

### Immunogold labeling

Ultrathin LR-White embedded sections, collected on Formvar carbon-coated nickel grids, were incubated in drops of 1% bovine serum albumin (BSA) in phosphate-buffered saline (PBS) containing 0.02 M glycine and normal goat serum at room temperature for 30 min [38]. Sections were then incubated overnight with a rabbit monoclonal anti-vimentin antibody (clone V9; Ventana, Tucson, AZ, USA; pre-diluted) at 4°C. After several washes with PBS + 0.1% BSA, grids were incubated with a 20 nm secondary antibody-gold particle complex (Agar Scientific, Stansted Essex CM24 8GF United Kingdom) at 1:10 diluted in PBS 0.1% BSA for 2 h at room temperature. After immunolabeling, sections were washed with PBS + 0.1% BSA, washed in distilled water, dried, and counterstained with uranyl acetate. All sections were examined with a Hitachi 7100 FA electron microscope.

### Statistical analysis

Statistical analysis was performed using GraphPad Prism 5 Software (La Jolla, CA, USA). Spatial distribution of microcalcifications within mammary lesions were analyzed by the Chi square test (*P*< 0.0001) to compare microcalcifications isotypes among BLm, ISCm, ICm and ICwm and by Fisher’s exact tests (*P*< 0.0001) to analyze the associations between pairs of data sets.

Immunohistochemical data were analyzed by Kruskal-Wallis test (*P*< 0.0001) and by Mann–Whitney test (*P*< 0.0005).

## Results

### Morphology

Samples were classified as follows: 22 benign lesions (14 fibrocystic mastopathies and eight fibroadenomas) with microcalcifications (BLm), 21 ductal *in situ* carcinomas with microcalcifications (ISCm), 23 infiltrating ductal carcinomas with microcalcifications (ICm) and 20 infiltrating ductal carcinomas without microcalcifications (ICwm).

With regard to the morphology of microcalcifications, we found birefringent crystals in 14 BLm (eight fibrocystic mastopathies and six fibroadenomas), psammoma bodies in eight malignant lesions (seven ISCm and one ICm), polymorphous bodies in both BLm (six fibrocystic mastopathies and two fibroadenomas) and malignant lesions (14 ISCm and 23 ICm) (see Additional file 1).

### Microcalcifications elemental analysis

**Figure 1:**
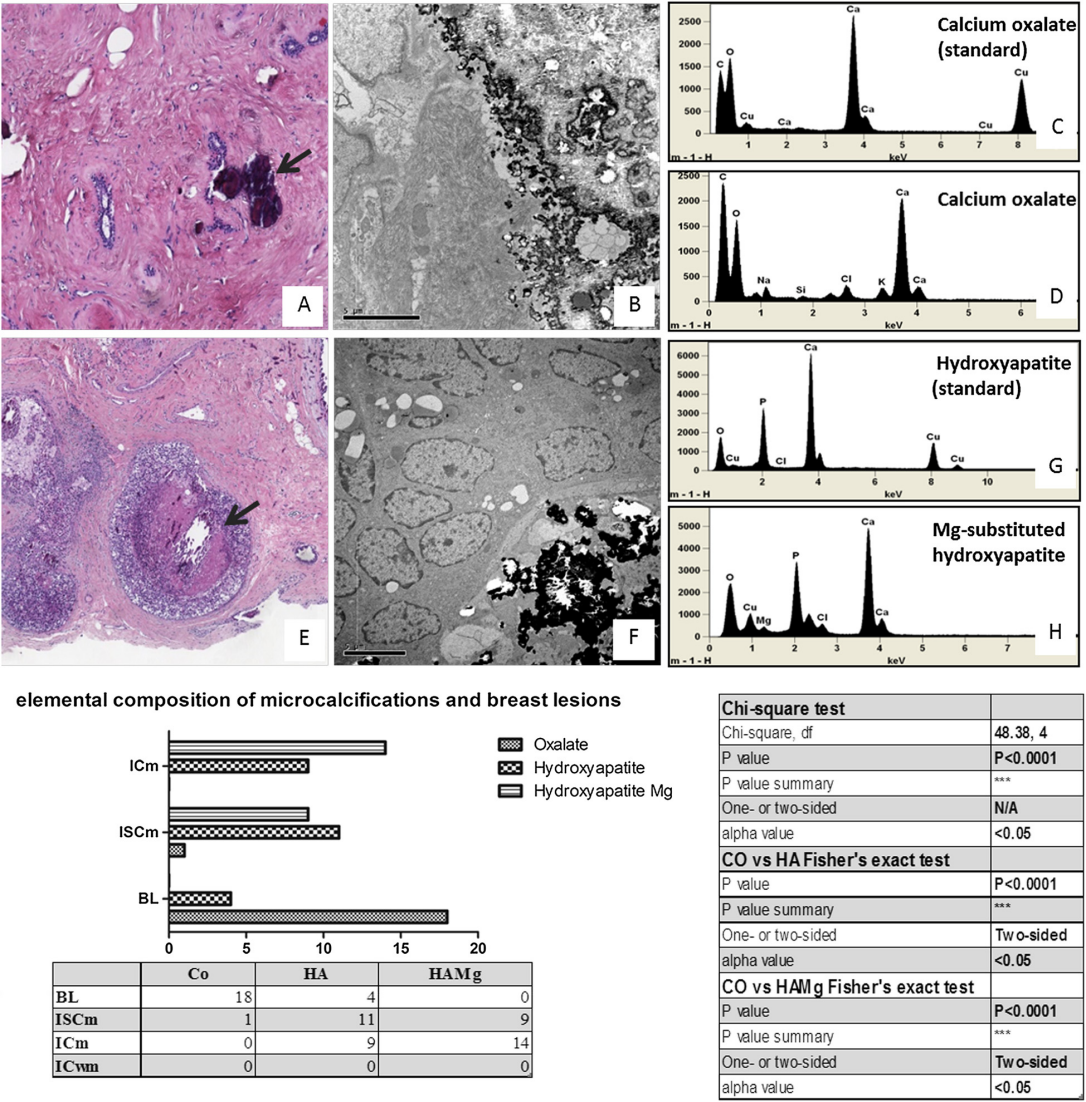
**Elemental composition of calcification in breast pathology. (A)** Microcalcifications (arrow) in BLm (fibroadenoma). **(B)** Electron micrograph by TEM of the microcalcification indicated in **(A)**. **(C)** EDX spectra obtained by microanalysis of commercial standard sample utilized as a control. **(D)** EDX spectrum revealed that microcalcifications were composed of calcium oxalate (CO). **(E)** Microcalcifications (arrow) in an ISCm (comedocarcinoma). **(F)** Electron micrograph by TEM of the microcalcification indicated in (E). **(G)** EDX spectra obtained by microanalysis of commercial standard sample utilized as a control. **(H)** EDX spectrum revealed that this microcalcification was composed of magnesium-substituted hydroxyapatite (Mg-Hap). **(I)** Microcalcification type related to breast pathology by statistical analysis.

The ultrastructural elemental microanalysis performed on breast microcalcifications confirmed the presence of the already-known types of calcifications, CO and HA (Figure 1). In particular, CO microcalcifications appeared as unstained birefringent crystals in 79% of cases and as polymorphous bodies in 21% of cases; among the 24 HA microcalcifications, we observed seven psammoma bodies and 17 polymorphous bodies, whereas most of the magnesium-substituted hydroxyapatite (Mg-HAp) microcalcifications appeared as polymorphous bodies (22 polymorphous bodies and one psammoma body).

The presence of CO correlated with benign lesions in 81.8% of cases (18 out of 22), whereas 97.7% (43 out of 44) of malignant lesions were characterized by the presence of complex forms of microcalcifications (Figure 1). For the first time, EDX microanalysis allowed us to identify a new subtype of complex HA form, Mg-HAp (Figure 1E,F and H). It is important to underline that Mg-HAp was detected only in malignant lesions (23 out of 44) whereas CO was never found in ICm (Figure 1).

### Epithelial cells undergoing mesenchymal transition

**Figure 2:**
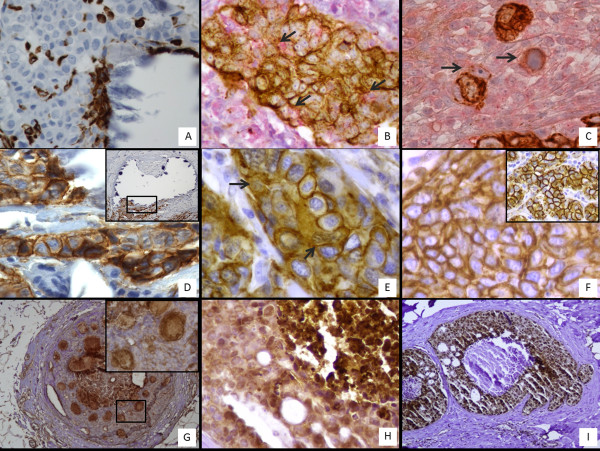
**Breast cancer microcalcifications and mesenchymal phenotype. (A)** Vimentin-positive cells in a ductal *in situ*comedocarcinoma in proximity of calcium deposits. **(B)** Double staining for pan-cytokeratin (brown stain) and vimentin (red cytoplasmic stain). The co-localization of both markers (arrows) highlight the EMT just as it is occurring. The same phenomenon was observed in cells infiltrating the stroma as small aggregates (arrows) **(C)**. Double-stain demonstrating keratin positivity differentiated these cells from stromal elements. β-Catenin immunostaining demonstrated the translocation of the signal in the cytoplasm/nucleus of cells close to a microcalcification in the Icm **(D** and **E)**. Notably, ICwm showed a prevalent β-catenin membrane stain **(F)**. The insert in **(F)** illustrates cell membrane positivity to β-catenin signal in normal breast tissue. **(G)** Image showing a strong signal for β 2-M near a microcalcification in ISCm. OPN signal **(H)** and BMP2 signal **(I)** in cells surrounding calcium deposits allowed us to assume that mineralization observed in breast is similar to that which occurs in bone.

**Figure 3:**
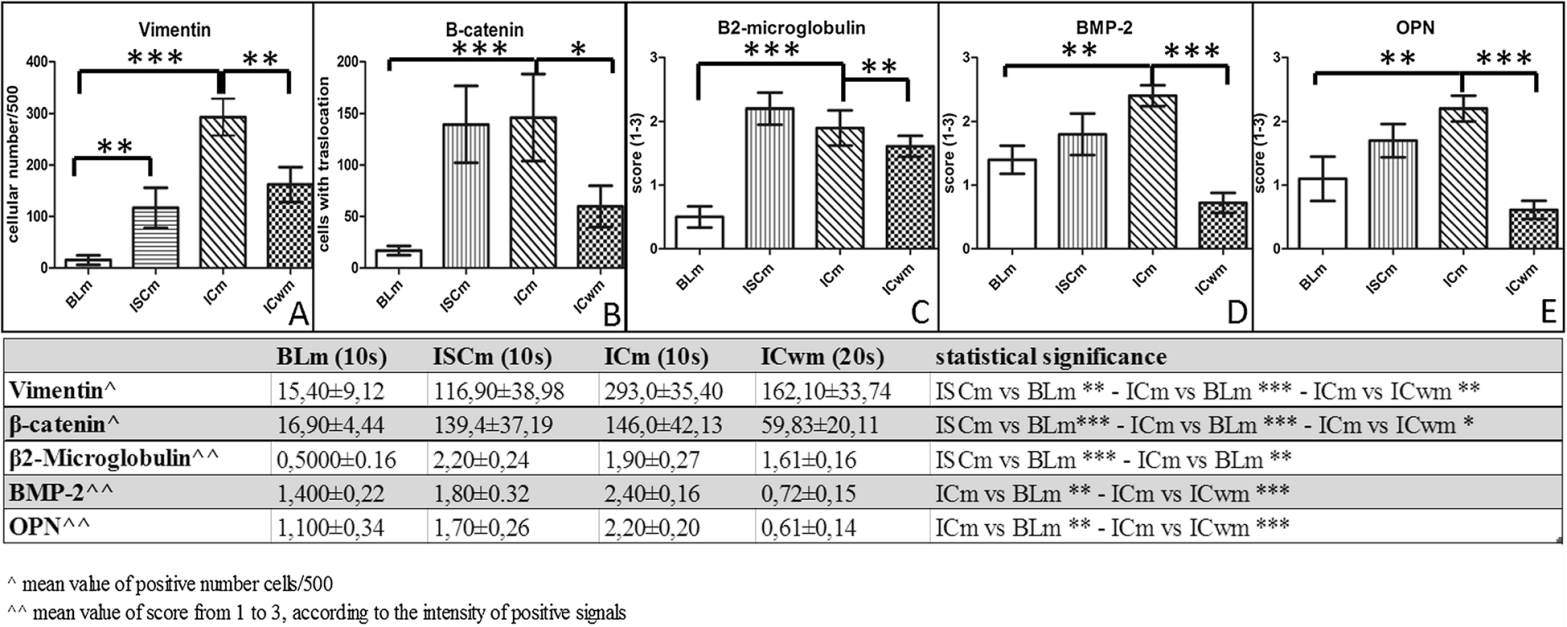
**Immunohistochemistry (IHC) to investigate mesenchymal characteristics and mineralization capability.** Quantification of mesenchymal marker expression. IHC for vimentin **(A)** and β-catenin **(B)** was evaluated by counting the number of positive cells up to a total of 500 for each sample in a randomly-selected area containing microcalcifications. β2-M **(C)**, BMP-2 **(D)** and OPN **(E)** were evaluated assigning a score from 1 to 3 according to the intensity of positive signals in randomly-selected regions containing microcalcifications. Immunohistochemical data are reported in the table; horizontal bars in the graphs represent significant differences (*P<0.05; **P<0.01; ***P< 0.001).

Mesenchymal characteristics were assessed by vimentin and β-catenin detection. Immunohistochemical reactions were evaluated by counting the number of positive cells up to a total of 500 for each sample in a randomly-selected area containing microcalcifications (Figure 2B–F). The rate of vimentin positive cells was significantly higher in malignant breast lesions with microcalcifications (293.0 ± 35.4 in ICm; 116.9 ± 38.9 in ISCm) as compared with BLm (15.4 ± 9.1) (Figure 3A).

Notably, we found that among infiltrating carcinomas, ICm showed a significantly higher number of vimentin-positive cells (293.0 ± 35.4) as compared with ICwm (162.1 ± 33.7) (Figure 3A). We found the same trend when studying the translocation of β-catenin from the cytoplasmic membrane to the cytoplasm and to the nucleus (Figure 2D, E and F). Interestingly, we detected a strong increase in cells showing cytoplasmic/nuclear β-catenin staining in malignant lesions with microcalcifications (ICm 146.0 ± 42.13 vs ICwm 59.83 ± 20.l1) (Figure 3B).

The dramatically different rate of cells with vimentin and nuclear β-catenin expression in ICm as compared with BLm and ICwm suggested that the formation of microcalcifications could be related to the EMT phenomenon (Figure 3A and B).

### Osteoblastic differentiation and mineralization

Reactions for β2-microglobulin (β2-M), bone morphogenic protein-2 (BMP-2) and osteopontin (OPN) were evaluated by assigning a score from 1 to 3 according to the intensity of positive signals in randomly-selected regions (Figure 2G,H and I). As reported in Figure 3C, our results showed a striking increase in β2-M signal in cancerous lesions with microcalcifications (2.0 ± 0.1) compared with both BLm (0.5 ± 0.1) and ICwm (1.5 ± 1.1). Moreover, we demonstrated a significant difference in BMP-2 expression between infiltrating carcinomas with (2.4 ± 0.1) or without microcalcifications (0.7 ± 0.1) (Figure 3D).

The signal of OPN appeared very low in ICwm and homogenously widespread in BLm with CO microcalcifications (Figure 3E). In contrast, OPN showed a focal distribution with an increase in the signal in the proximity of HA and Mg-HAp microcalcifications (Figure 2H).

### Osteoblast-like cell characterization

**Figure 4:**
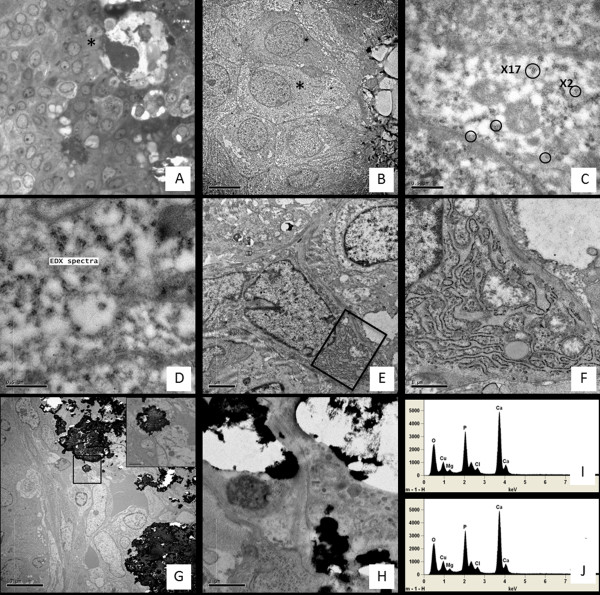
**Osteoblast like-cell identification.** Ultrastructural analysis of breast cancer cells surrounding microcalcifications showed some elongated cells (LR-White embedding). **(A)** Toluidine blue stain on a semi-thin section of cells surrounding microcalcification in malignant lesions. **(B)** Electron micrograph obtained by TEM of cells shown in **(A)**. **(C)** Ultrastructural details of cells obtained by immunogold reaction for vimentin. Numerous gold particles indicated the presence of vimentin filaments inside the cytoplasm of this cell (circle). **(D)** Electron micrograph obtained by TEM on electron-dense intracytoplasmic bodies in the analyzed cell. EDX spectrum confirmed these consisted of HA crystals **(I). (E)**, **(F)**, **(G)** and **(H)** present finer ultrastructural analysis of breast cancer cells surrounding microcalcifications, as performed on Epon-embedded tissue. **(E)** and **(F)** show ultrastructural details of a cell exhibiting osteoblast-like morphology with a large cytoplasm and a huge rough reticulum. **(G)** Cell near a microcalcification containing a HA-matrix vesicle. The enlargement in **(G)** and **(H)** capture a matrix vesicle just as it initiated the exocytosis of HA (EDX spectrum in **(I)** and **(J)**).

Our transmission electron microscopy study of cells located near HA and Mg-HAp microcalcifications revealed the presence of cells with morphological characteristics typical of osteoblasts (Figure 4). Osteoblast like-cells identified surrounding calcium deposits were positive for vimentin, as shown by immunogold labeling (Figure 4A, B and C). Their cytoplasms were rich in vesicles containing electron-dense granules similar to the intracellular vesicle of the osteoblasts (Figure 4G and H; these intracellular vesicles were secreted outside cells (Figure 4G and H). Elemental analysis of the electron-dense bodies inside these vesicles demonstrated the presence of HA (Figure 4I and J).

## Discussion

Microcalcifications have a crucial role in breast cancer diagnosis. However, the mechanisms that induce their formation are still unknown [8]. In this paper, we investigated breast microcalcifications and hypothesized that they could result from a mineralization process similar to that of bone osteogenesis, sustained by the EMT phenomenon induced by microenvironmental stimulatory factors.

In our samples, we detect psammoma bodies in well-differentiated carcinomas and polymorphous bodies both in ISCm and in ICm, as previously described [39]. Our data regarding the elemental composition of microcalcifications not only confirmed previous data about the presence of CO and HA [5-7] but most importantly allowed us to describe for the first time the presence of Mg-HAp in breast microcalcifications. It is important to underline that the complex forms of calcification (HA and Mg-HAp) are strictly related to malignant lesions whereas CO is mainly reported in benign lesions. Surprisingly, the EDX analysis revealed that some microcalcifications detected by light microscopy as polymorphous bodies were made of CO. We postulated that this morphology could be due to a protein coat on the CO crystal. Mg-HAp was not found in benign lesions whereas it was frequently detected in breast cancer. The capability of HA to bind to bicationic ions such as Mg [40], may confer carcinogenic properties on HA since a Mg-depleted microenvironment can influence the DNA repair processes and the control of proliferation and apoptosis [41,42]. The EDX data allowed us to hypothesize an active role of microcalcifications in breast carcinogenesis, since such complex microcalcifications cannot be due to a mere degenerative process but rather resemble the physiological process of mineralization that occurs in bone. At the same time, the presence of complex forms of calcification raises an interesting question: how can breast epithelial cells produce HA? Recently, Cox *et al.*[9,43] investigated the molecular mechanisms of the microcalcification process in breast cell cultures and demonstrated that the mineralization process, related to alkaline phosphatase activity, could be similar to that observed in bone matrix formation.

**Figure 5:**
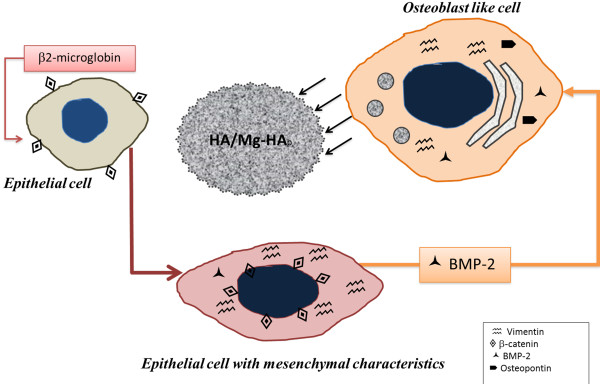
**Model for mesenchymal transformation and calcium deposition in breast cancer.** Physiological bone mineralization involves mesenchymal cells expressing vimentin and cytoplasmic/nuclear β-catenin. In our hypothesis, epithelial cells acquire mesenchymal characteristics in a microenvironment conditioned by β2-microglobulin. Under BMP-2-induction, epithelial cells that have acquired the mesenchymal phenotype could assume an osteoblast-like phenotype and behave as a producer of complex forms of calcification.

In response to these data, we investigated if the acquisition of mesenchymal characteristics could be a junction ring between breast epithelial cells and the complex microcalcifications that we described in breast cancer (Figure 5). Numerous studies have reported the EMT phenomenon in breast lesions [19,21] even though, to the best of our knowledge, no correlation of this phenomenon to calcium deposition has been made to date. Our data on mesenchymal markers (i.e. vimentin and β-catenin translocation) provided evidence that epithelial cells acquire mesenchymal characteristics in a process of mineralization in breast cancer. Indeed, the number of vimentin-positive cells was dramatically different in infiltrating carcinomas with or without microcalcifications.

The results obtained by dual color immunohistochemistry for cytokeratin and vimentin markers further supported the evidence of mesenchymal transformation in ICm, since we observed cells in transition expressing both markers. In addition, these data were also sustained by the slight β-catenin translocation from the cytoplasmic membrane to cytoplasm and nuclei [44]. Furthermore, we examined some of the most important factors able to trigger the EMT. In particular, β2-M was reported as a growth factor and signaling molecule in cancer cells [45-47]. It is also known to be able to trigger the EMT phenomenon as well as being capable of activating stromal cells, such as osteoblasts [48] and osteoclast [49]. The significant increase in this molecule in our breast cancer samples with microcalcifications suggests its possible role both in mesenchymal transformation and in the production of microcalcifications. To strengthen these data, we studied molecules involved in physiological bone mineralization. BMP2 is a member of the transforming growth factor superfamily and able to induce mineralization in osteoblasts cultures [50,51]. Furthermore, Liu *et al.* recently demonstrated a correlation between serum levels of BMP2 and breast microcalcifications [52]. Our results for the expression of BMP-2 in tissues with microcalcifications allowed us to assimilate the mineralization observed in the lesions of mammary glands with that occurring in osteoblast cultures, since both respond to the same signal.

Strikingly, when comparing *in situ* and infiltrating carcinomas with microcalcifications the signal appears to be intensively localized in the microenvironment surrounding the microcalcifications. OPN is another molecule that plays an important role in the mineralization process since it regulates both HA production [53] and its inhibition [54] depending on its phosphorylation state [55]. The data reported here on the expression of OPN suggest that it plays a role quite similar to that exerted during the physiological process of mineralization in bone [56,57].

Thus, such mineralization phenomenon in the context of the microcalcifications suggests the existence of cells able to produce HA. To provide proof of this conjecture, we performed an ultrastructural study in an attempt to identify cells with an osteoblast-like phenotype. At the ultrastructural level, the osteoblast is characterized by the presence of a well-developed rough endoplasmic reticulum with dilated cisternae and a dense granular content and by a large circular Golgi complex comprising multiple Golgi stacks [56]. The morphological characterization of cells surrounding the mineralized core displayed numerous cells exhibiting a mesenchymal phenotype surprisingly similar to osteoblasts. These osteoblast-like cells presented electron-dense bodies in their cytoplasmic vesicles. The elemental characterization of these vesicles demonstrated that their content consisted of HA, a typical feature of osteoblast intracellular vesicles [58,59]. Taken together, these evidence led us to hypothesize that intracellular vesicles could be referred to as the center of nucleation of HA. Notably, we frequently observed osteoblast like cells secreting HA into the extracellular space. Finally, we confirmed by ultrastructural immunohistochemistry that these cells have a mesenchymal phenotype, as verified by their positivity to vimentin.

Although the phenomenon of breast microcalcifications could be sustained by several mechanisms, the finding of osteoblast-like cells led us to hypothesize that microcalcifications in breast lesions could represent an active process related to epithelial cells with mesenchymal characteristics.

## Conclusions

New insights into the complex phenomenon of breast microcalcification could better define the pathophysiology of different microcalcifications. The introduction of mesenchymal markers such as vimentin and elemental analysis of breast lesions with microcalcifications may add further data to complete the clinical setting in the diagnosis and care of patients. The finding of a specific elemental composition associated with microcalcifications in cancer could enhance imaging technologies to discriminate microcalcifications *in vivo*, and thus act as a helpful tool in breast cancer screening.

## Abbreviations

BLm: Benign lesion with microcalcifications; BMP-2: Bone morphogenic protein; CO: Calcium oxalate; DAB: Diaminobenzidine; EDX: Energy dispersive x-ray; EMT: Epithelial to mesenchymal transition; H & E: Hematoxylin and eosin; HA: Hydroxyapatite; HAMg: Hydroxyapatite magnesium substituted; ICm: Infiltrating carcinoma with microcalcifications; ICwm: Infiltrating carcinoma without microcalcifications; IHC: Immunohistochemistry; ISCm: *In situ* carcinoma with microcalcifications; Mg: Magnesium; OPN: Osteopontin; TMA: Tissue Micro-Array; β2-M: β2-microglobulin.

## Competing interests

The authors declare that they have no competing interests.

## Authors’ contributions

MS carried out the electron microscopy studies (EDX and colloidal gold studies), participated in the immunohistochemistry and drafted the manuscript. EG carried out the case selection and morphological classification. CA carried out immunohistochemistry. CAP participated in the design of the study and cases selection. LGS participated in the design of the study and cases selection. EB conceived of the study, and participated in its design, overall review of the result and coordination and helped to draft the manuscript. All authors were involved in writing the paper and had final approval of the submitted and published versions.

## Pre-publication history

The pre-publication history for this paper can be accessed here:

http://www.biomedcentral.com/1471-2407/14/286/prepub

## Supplementary Material

Additional file 1 **Descriptive classification of microcalcifications in benign and malignant breast lesions.**http://www.biomedcentral.com/imedia/5099083231233250/supp1.pdf. Click here for file
