# Supplementary material for: Microcalcifications in breast cancer: an active phenomenon mediated by epithelial cells with mesenchymal characteristics
Source: BMC Cancer. 2014 Apr 23;14:286. doi: 10.1186/1471-2407-14-286 (PMC4021315; doi:10.1186/1471-2407-14-286)
Supplement: Additional file 1 — Descriptive classification of microcalcifications in benign and malignant breast lesions.http://www.biomedcentral.com/imedia/5099083231233250/supp1.pdf. [file 1471-2407-14-286-S1.pdf]

Sample	Histological diagnosis	Microcalcification type	Morphological aspect
BL1	fibrocystic mastopaties	calcium oxalate	birefrigent crystal
BL2	fibrocystic mastopaties	calcium oxalate	birefrigent crystal
BL3	fibrocystic mastopaties	hydroxyapatite	polymorphous body
BL4	fibrocystic mastopaties	hydroxyapatite	polymorphous body
BL5	fibrocystic mastopaties	calcium oxalate	birefrigent crystal
BL6	fibrocystic mastopaties	calcium oxalate	polymorphous body
BL7	fibrocystic mastopaties	calcium oxalate	birefrigent crystal
BL8	fibrocystic mastopaties	hydroxyapatite	polymorphous body
BL9	fibrocystic mastopaties	calcium oxalate	birefrigent crystal
BL10	fibrocystic mastopaties	calcium oxalate	polymorphous body
BL11	fibrocystic mastopaties	calcium oxalate	birefrigent crystal
BL12	fibrocystic mastopaties	calcium oxalate	birefrigent crystal
BL13	fibrocystic mastopaties	calcium oxalate	birefrigent crystal
BL14	fibrocystic mastopaties	calcium oxalate	polymorphous body
BL15	fibroadenomas	hydroxyapatite	polymorphous body
BL16	fibroadenomas	calcium oxalate	birefrigent crystal
BL17	fibroadenomas	calcium oxalate	birefrigent crystal
BL18	fibroadenomas	calcium oxalate	birefrigent crystal
BL19	fibroadenomas	calcium oxalate	birefrigent crystal
BL20	fibroadenomas	calcium oxalate	polymorphous body
BL21	fibroadenomas	calcium oxalate	birefrigent crystal
BL22	fibroadenomas	calcium oxalate	birefrigent crystal
ISCm1	DCIS cribiform type	hydroxyapatite	polymorphous body
ISCm2	DCIS cribiform type	hydroxyapatite	psammoma body
ISCm3	DCIS cribiform type	hydroxyapatite Mg	psammoma body
ISCm4	DCIS cribiform type	calcium oxalate	polymorphous body
ISCm5	DCIS cribiform type	hydroxyapatite Mg	polymorphous body
ISCm6	DCIS cribiform type	hydroxyapatite	polymorphous body
ISCm7	DCIS cribiform type	hydroxyapatite Mg	polymorphous body
ISCm8	DCIS cribiform type	hydroxyapatite	polymorphous body
ISCm9	DCIS cribiform type	hydroxyapatite Mg	polymorphous body
ISCm10	DCIS comedo type	hydroxyapatite	psammoma body
ISCm11	DCIS comedo type	hydroxyapatite	psammoma body
ISCm12	DCIS comedo type	hydroxyapatite Mg	polymorphous body
ISCm13	DCIS comedo type	hydroxyapatite Mg	psammoma body
ISCm14	DCIS comedo type	hydroxyapatite	polymorphous body
ISCm15	DCIS comedo type	hydroxyapatite	polymorphous body
ISCm16	DCIS comedo type	hydroxyapatite	polymorphous body
ISCm17	DCIS comedo type	hydroxyapatite Mg	polymorphous body
ISCm18	DCIS comedo type	hydroxyapatite	psammoma body
ISCm19	DCIS comedo type	hydroxyapatite Mg	polymorphous body
ISCm20	DCIS comedo type	hydroxyapatite Mg	polymorphous body
ISCm21	DCIS micropapillary type	hydroxyapatite	psammoma body
ICm1	invasive duct carcinoma	hydroxyapatite Mg	polymorphous body
ICm2	invasive duct carcinoma	hydroxyapatite	polymorphous body
ICm3	invasive duct carcinoma	hydroxyapatite Mg	polymorphous body
ICm4	invasive duct carcinoma	hydroxyapatite Mg	polymorphous body
ICm5	invasive duct carcinoma	hydroxyapatite	polymorphous body
ICm6	invasive duct carcinoma	hydroxyapatite	polymorphous body
ICm7	invasive duct carcinoma	hydroxyapatite Mg	polymorphous body
ICm8	invasive duct carcinoma	hydroxyapatite	psammoma body
ICm9	invasive duct carcinoma	hydroxyapatite Mg	polymorphous body
ICm10	invasive duct carcinoma	hydroxyapatite	polymorphous body
ICm11	invasive duct carcinoma	hydroxyapatite Mg	polymorphous body
ICm12	invasive duct carcinoma	hydroxyapatite Mg	polymorphous body
ICm13	invasive duct carcinoma	hydroxyapatite	polymorphous body
ICm14	invasive duct carcinoma	hydroxyapatite Mg	polymorphous body
ICm15	invasive duct carcinoma	hydroxyapatite Mg	polymorphous body
ICm16	invasive duct carcinoma	hydroxyapatite	polymorphous body
ICm17	invasive duct carcinoma	hydroxyapatite Mg	polymorphous body
ICm18	invasive duct carcinoma	hydroxyapatite Mg	polymorphous body
ICm19	invasive duct carcinoma	hydroxyapatite	polymorphous body
ICm20	invasive duct carcinoma	hydroxyapatite Mg	polymorphous body
ICm21	invasive duct carcinoma	hydroxyapatite Mg	polymorphous body
ICm22	invasive lobular carcinoma	hydroxyapatite	polymorphous body
ICm23	invasive lobular carcinoma	hydroxyapatite Mg	polymorphous body
